# Multi-Walled Carbon Nanotubes Promote Cementoblast Differentiation and Mineralization through the TGF-β/Smad Signaling Pathway

**DOI:** 10.3390/ijms16023188

**Published:** 2015-02-02

**Authors:** Lu Li, Zhimin Zhu, Weixiong Xiao, Lei Li

**Affiliations:** State Key Laboratory of Oral Diseases, Department of Prosthodontics, West China Hospital of Stomatology, Sichuan University, Chengdu 610041, China; E-Mails: 2013324030048@stu.scu.edu.cn (L.L.); zzhimin@163.com (Z.Z.); zhangao0917@gmail.com (W.X.)

**Keywords:** carbon nanotubes, cementoblasts, differentiation, mineralization

## Abstract

Excretion of cementum by cementoblasts on the root surface is a process indispensable for the formation of a functional periodontal ligament. This study investigated whether carboxyl group-functionalized multi-walled carbon nanotubes (MWCNT-COOH) could enhance differentiation and mineralization of mammalian cementoblasts (OCCM-30) and the possible signaling pathway involved in this process. Cementoblasts were incubated with various doses of MWCNT-COOH suspension. Cell viability was detected, and a scanning electron microscopy (SEM) observed both the nanomaterials and the growth of cells cultured with the materials. Alizarin red staining was used to investigate the formation of calcium deposits. Real-time PCR and western blot were used to detect cementoblast differentiation and the underlying mechanisms through the expression of the osteogenic genes and the downstream effectors of the TGF-β/Smad signaling. The results showed that 5 µg/mL MWCNT-COOH had the most obvious effects on promoting differentiation without significant toxicity. *Alp*, *Ocn*, *Bsp*, *Opn*, *Col1* and *Runx2* gene expression was up-regulated. *Smad2* and *Smad3* mRNA was up-regulated, while *Smad7* was first down-regulated on Day 3 and later up-regulated on Day 7. The elevated levels of phospho-Smad2/3 were also confirmed by western blot. In sum, the MWCNT-COOH promoted cementoblast differentiation and mineralization, at least partially, through interactions with the TGF-β/Smad pathway.

## 1. Introduction

Cementum is the bone-like tissue covering the external surface of tooth root in a thin layer [1]. Due to its intermediary position, cementum forms the interface between root dentin and periodontal ligament. In other words, cementum is a part of the tooth, but functionally, it belongs to the dental attachment apparatus [2]. The integrity of cementum could be affected by bacterial endotoxin deposition in periodontitis, and the diseased cementum is often eliminated during periodontal therapy [3]. Therefore, one main goal of periodontal therapy is to form new cementum and to restore periodontal attachment. Although the biochemical composition of bone and cementum shows a notable similarity, cementum differs from bone by lacking innervation and vascularization, thus it has limited remodeling potential [1,4]. Since the extracellular matrix (ECM) regulates the cell response to environmental signals, the local environment of the matrix is of great importance in order to maintain the homeostasis of cementum. However, the matrix generated during healing from periodontal disease could cause altered structural integrity and biochemical composition, largely different from that of the healthy case. Therefore, in order to achieve new cementum and attachment regeneration rather than fibrous scar repair, the local environment must be conducive to the recruitment and function of cementum-forming cells [3]. Current approaches to solve this problem include the application of barrier membranes for guided tissue regeneration, growth factors and enamel matrix derivative (EMD) to root surfaces [5,6]. However, there are concerns for these therapies, such as high cost, rapid clearance and poor mechanical properties. Additionally, their effectiveness on new cementum and attachment formation is still unpredictable.

In micropatterning cellular studies, cells interact with extracellular matrix at the nanoscale [7,8]. The applications of biomaterials with nanotopography have been shown to create an environment favorable for cell attachment, proliferation and differentiation [9,10]. Carbon nanotubes (CNTs) were first discovered by Dr. Sumio Iijima in 1991 [11]. Their unique physiochemical properties, such as extremely small size, light weight, chemical and thermo-dynamical stability and outstanding mechanical properties, provide the potential for a broad range of medical applications [12]. Multi-walled carbon nanotubes (MWCNTs) are formed by rolling multiple sheets of graphene into a cylinder. CNTs, with a size comparable to extracellular matrix molecules, such as collagens and laminins, have been reported to sustain cell growth and bone formation in osteoblasts [10,13]. However, their poor solubility in aqueous solutions remains a major impediment in biological and medical applications. The recently developed chemical modification and functionalization have enabled CNTs to dissolve and disperse in water, making them more suitable for biological and medical applications [14,15].

Facing the high prevalence of periodontal disease and the limitations of the currently employed therapeutics, it is of great interest to investigate other techniques for enhancing cementogenesis. One conceivable method could be the application of CNTs to cementoblasts. There is no report about the biological effects of MWCNTs on cementoblasts and the promotion of cementogenesis. The purpose of this study, therefore, was to investigate the effect of* in vitro* functionalized MWCNTs (MWCNT-COOH, pristine carboxyl group-functionalized MWCNTs), cultured with cementoblasts to form a favorable microenvironment for cementogenesis, on cell proliferation, morphology, mineralization and the underlying molecules and mechanisms.

## 2. Results

### 2.1. Cytotoxicity of Carboxyl Group-Functionalized Multi-Walled Carbon Nanotubes (MWCNT-COOH)

**Figure 1:**
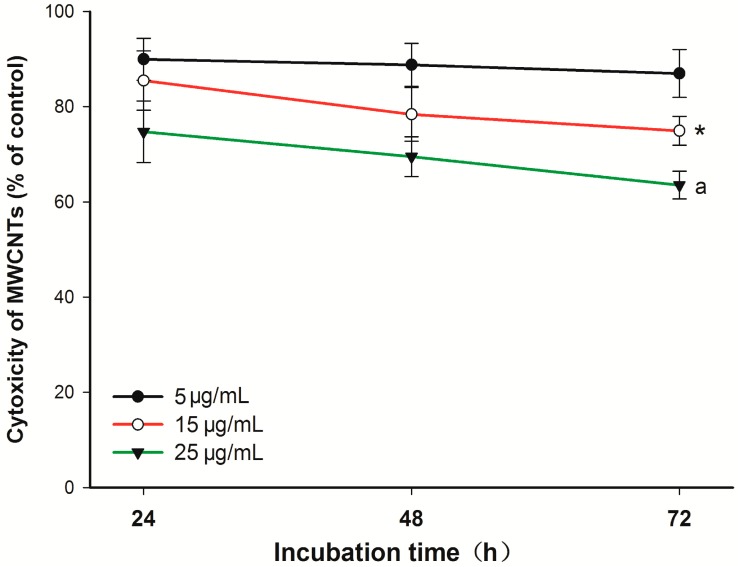
Cytotoxicity of carboxyl group-functionalized multi-walled carbon nanotubes (MWCNT-COOH) on cementoblasts at various concentrations after three days of incubation. Data are expressed as the mean ± SD. * *p* < 0.05, for 15 µg/mL MWCNT-COOH cells* vs.* control cells; ^a^ *p* < 0.001, for 25 µg/mL MWCNT-COOH cells* vs.* control cells.

The Cell Counting Kit-8 (CCK-8) assay demonstrated a dose- and time-dependent toxic potential of MWCNT-COOH on the viability of cementoblasts (Figure 1). The toxic effects of MWCNT-COOH on the cell viability were significant at higher concentrations (larger than 15 µg/mL) and after 48 h of incubation. Nevertheless, the cell viability of the 5 µg/mL group with 72 h of incubation was not significantly different from that of the control group.

### 2.2. Assessments of the Material Structure and Cell Growth

**Figure 2:**
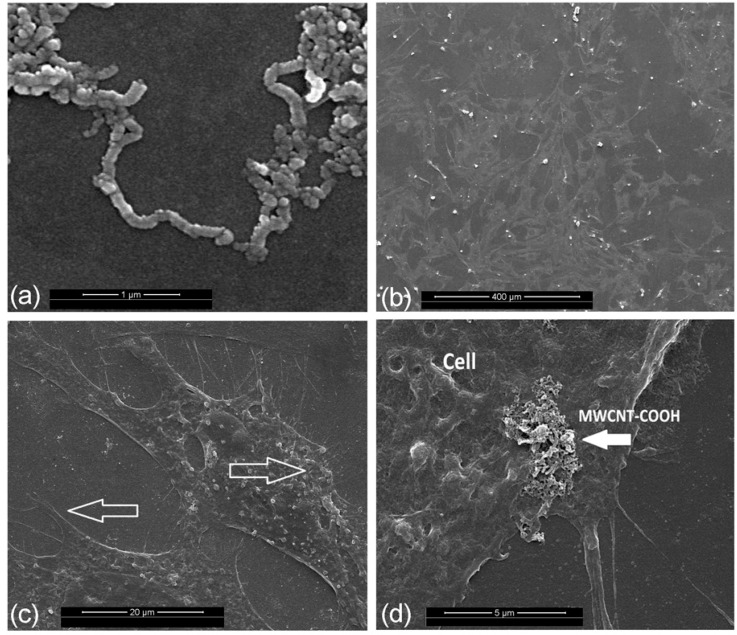
Representative images showing the characteristic features of MWCNT-COOH (**a**) and cementoblasts cultured with it (**b**–**d**): (**b**) cells were able to adhere and spread well in all directions with normal morphology; (**c**) numerous filopodia and microvillosities were extended from the cells; (**d**) higher magnifications show cell attachment to clusters of MWCNT-COOH.

SEM observations of MWCNT-COOH and cell morphologies are shown in Figure 2. The micrograph showed swirled carbon nanotubes without other amorphous or graphitic carbon nanostructures (Figure 2a). The twists were found periodically in the carbon nanotubes due to the functionalization via carboxylation processes. The surface of the carbon nanotubes is not smooth, but rough, which is due to shortening and defects in the functional site. In addition, cells were able to adhere and spread well in all directions with numerous cytoplasmic extensions on the MWCNT-COOH (Figure 2b–d). At higher magnifications, filopodia and microvillosities were noticeably extended from the cells toward the inside of the materials.

### 2.3. Effect of MWCNT-COOH on the Mineralization of Cementoblasts

**Figure 3:**
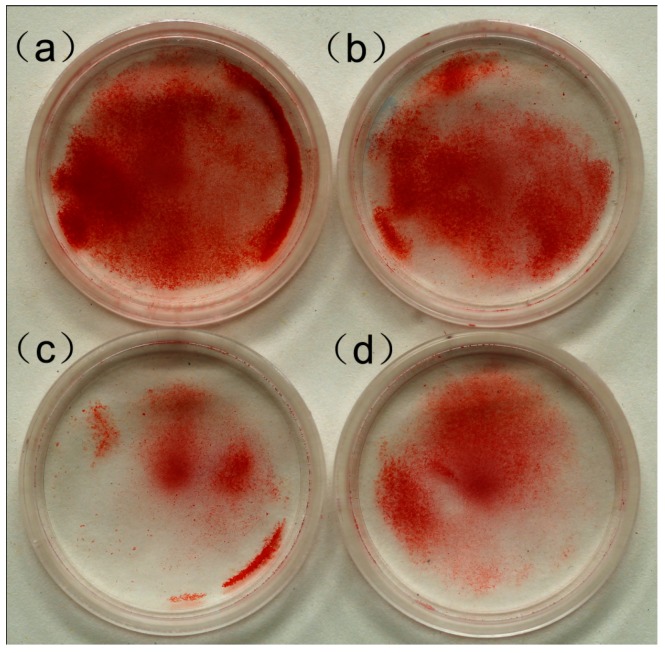
The alizarin red staining of the calcified nodules of cementoblasts after 14 days cultured in various concentrations of MWCNT-COOH: (**a**) 5 µg/mL; (**b**) 15 µg/mL; (**c**) 25 µg/mL; and (**d**) control.

Upon 14 days of incubation, red-stained mineralized nodules (an intercellular, round, light, tight mass with red staining) were clearly observed from the control group and treated groups (MWCNT-COOH 5, 15, 25 µg/mL), shown in Figure 3. Moreover, we found that the 5 µg/mL group had the most significant mineral nodule formation, while the 25 µg/mL group had the least.

#### 2.3.1. Effects of MWCNT-COOH on OCCM-30 Cementoblast Gene Expression: Dose Response

**Figure 4:**
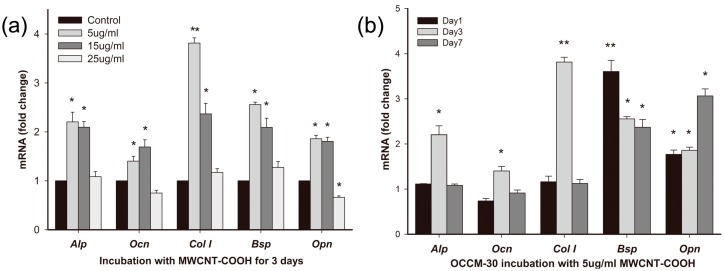

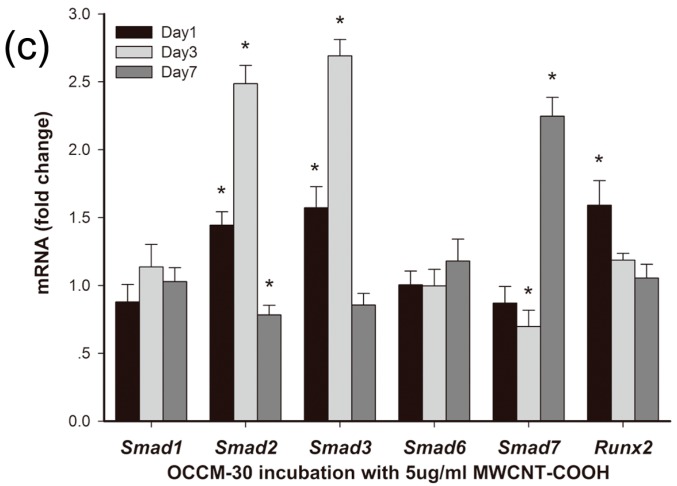
Results of gene expression. (**a**) Expression of genes associated with differentiation and mineralization of cementoblasts exposed to various concentrations of MWCNT-COOH; (**b**) time-course of the gene expression of cementoblasts in response to 5 µg/mL MWCNT-COOH; (**c**) expression of the runt-related transcription factor 2 (*Runx2*) and *Smad* genes of cementoblasts in response to 5 µg/mL MWCNT-COOH. Data are shown as the mean ± SD (* *p* < 0.05; ** *p* < 0.001).

The study showed that genes associated with differentiation and mineralization (alkaline phosphatase (*Alp*), osteocalcin (*Ocn*), bone sialoprotein (*Bsp*), osteopontin (*Opn*), type I collagen (*Col1*)) were differently affected by the three concentration levels of MWCNT-COOH (*i.e*., 5, 15 and 25 µg/mL) for the 72 h of incubation (Figure 4a). The dose of 5 µg/mL MWCNT-COOH was effective at eliciting cell responses. Significant upregulation in the 5 µg/mL MWCNT-COOH group was observed in *Alp* (2.2-fold of the control), *Col1* (3.8-fold), *Bsp* (2.56-fold) and *Opn* (1.86-fold), while 15 µg/mL MWCNT-COOH enhanced *Ocn* (1.69-folds) expression. However, the 25 µg/mL MWCNT-COOH failed to upregulate the aforementioned gene expression.

#### 2.3.2. Effects of MWCNT-COOH on OCCM-30 Cementoblast Gene Expression: Time Course

Having established the osteogenic genes upregulated by MWCNT-COOH mentioned above, we then moved forward to detect the time needed for realizing these regulatory effects. A dose of 5 µg/mL MWCNT-COOH was chosen for the next tests, because this dose caused an obvious response, but without significant cytotoxicity for the genes of interest. Our time course (1, 3 and 7 days) experiments showed that these genes responded quickly to MWCNT-COOH exposure within three days of incubation (Figure 4b): *Alp*, *Ocn*, *Bsp* and *Col1* mRNA expression reached peaks within three days, while *Opn* mRNA expression was up-regulated (three-fold of the control) on Day 7.

To better understand the underlying mechanism, we further investigated the gene expression encoded in the TGF-β/Smad pathway, as well as a key regulator of differentiation and function, namely Runt-related transcription factor 2 (*Runx2*) (Figure 4c). Compared to the control, a dose of 5 µg/mL MWCNT-COOH was able to upregulate the expression of the *Runx2* gene (1.59-times) in the differentiation and mineralization of cementoblasts on Day 1 and then gradually decreased with time. A significantly higher expression of the Smad2 gene was found on Day 1 (1.44-times the control) and Day 3 (2.49-times). At the end of the experiment (day 7), *Smad2* gene expression declined to a lower grade of 0.78-times. The expression of the *Smad3* gene had a similar trend, which increased from 1.57-times on Day 1 to 2.69 on Day 3 and then declined to 0.86-times on Day 7. Contrarily, expression of the *Smad1* and *Smad6* genes was not significantly changed in response to the MWCNT-COOH treatments. *Smad7* expression was significantly activated (2.25-times) on Day 7.

### 2.4. Effects of MWCNT-COOH on OCCM-30 Cementoblast Smad Proteins

**Figure 5:**
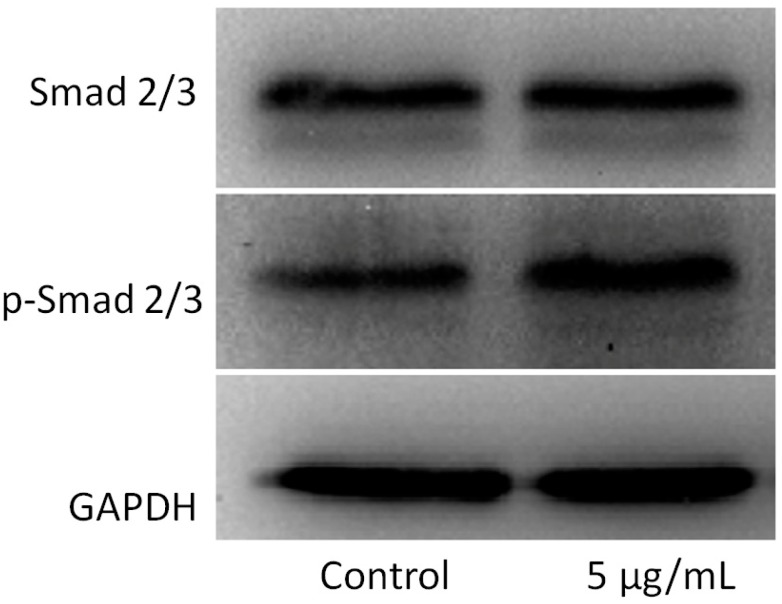
Effects of MWCNT-COOH on Smad proteins by western blot. On Day 3, increased p-Smad2/3 was observed in cementoblasts treated with 5 µg/mL MWCNT-COOH compared to the control.

Typically, the activation of the TGF-β/Smad signaling pathway mediates the phosphorylation of Smad2/3, which dimerizes with Smad4 and translocates to the nucleus, where they induce specific gene transcription [16,17]. Therefore, we analyzed the levels of p-Smad2/3 by western blot. On Day 3, an increased p-Smad2/3/Smad2/3 ratio was observed in OCCM-30 cementoblasts treated with 5 µg/mL MWCNTs compared to the control (Figure 5).

## 3. Discussion

In periodontics, cementum regeneration is crucial for the restoration of attachment loss in order to achieve functional periodontium. Studies have emphasized the significance of restoring or providing an appropriate micro-environment to initiate and promote new cementum formation [3]. The application of CNTs as a biomaterial has previously been debated in the literature. Some studies have reported that the cytotoxicity of certain nanomaterials and additional adverse effects might be associated with CNTs [18,19,20]. However, the functionalization process can improve CNTs’ processability and biocompatibility [21]. The modification of the chemical surface of nanotube structures with various functional groups increases their solubilization. When uniformly distributed in aqueous media, nanotubes can be internalized and translocated inside the cell [22]. Dr. Dandan Liu reported that toxic effects can be induced by the presence of 30 µg/mL carboxylated carbon nanotubes in mesenchymal stem cells [23]. For this reason, we examined MWCNT-COOH at three different concentrations (*i.e*., 5, 15 and 25 µg/mL) in order to determine a toxicological threshold for cementoblasts. In the present study, the low concentration of MWCNT-COOH (5 µg/mL) showed no significant cytotoxicity during the whole incubation. The filopodial extensions following the MWCNTs indicated a high affinity between cells and MWCNT-COOH. These observations are in agreement with the good proliferation of MC3T3-E1 cells on MWCNTs in a previous study [24].

Using alizarin red staining, the mineralized nodules in the MWCNT-COOH group were larger in number and greater in size as compared to those of the control, indicating the potential of nanomaterials to promote cementoblast mineralization. For one thing, the exceptionally rough surface of the carbon nanotubes’ topography could provide an enhancement of osteoblastic adhesion. In addition, the size of the CNTs’ diameters is close to that of collagen triple-helix, indicating that it may function like collagen fibrils to exert control over crystal nucleation and growth of inorganic components [25].

Besides the above observations, the underlying molecular process was also investigated. The expression of genes associated with OCCM-30 mineralization was first tested at various concentrations of MWCNT-COOH, and the most efficient dose (5 µg/mL) was further tested at various incubation times. We observed significantly up-regulated *Bsp*, *Alp*, *Ocn* and *Col1* gene expression compared to the control within 72 h, indicating a relatively quick response to MWCNT-COOH regulation. Additionally, up-regulation of *Opn* mRNA expression reached a peak at Day 7. *Alp* is one of the membrane-associated enzymes, while *Bsp*, *Opn*, *Ocn* and *Col1* belong to bone matrix proteins. These macromolecules together are involved in the regulation of cell mineralization. It is worth noting that *Opn* is a negative regulator of mineralization, which inhibits the growth of mineral crystals, a late stage of osteogenic differentiation [26]. Together, these results suggested that once mature cementoblasts have fulfilled their role in the cementum formation, they may move to the next stage of maintaining periodontal homeostasis by self-regulating their mineralization capacity.

Similar to bone regeneration, cementogenesis is a process closely related to numerous factors: transcriptional factors and growth factors [27]. Previous studies have shown that the TGF-β superfamily induced greater alveolar bone regeneration and cementogenesis in a variety of animal models [28,29,30,31]. TGF-β elicits its effect on cementogenesis by activating their receptors to induce phosphorylation of a group of intracellular transcription factors, known as Smads [32]. The activated Smad complex can then move into the nucleus, where it participates in the transcription of a set of target genes. Till now, researchers have demonstrated the effects of carbon nanotubes on various types of cells and different cell signaling pathways, among which the Smad proteins were shown to be activated in these nano-cell interactions [33]. Runt-related transcription factor 2 (*Runx2*, also called Cbfa1, AML3 or OSF2) belongs to the Runx family and interacts with Smads. Runx2 is the earliest osteogenic marker that binds to the osteocalcin promoter and is essential for bone development [34,35,36]. Therefore, we hypothesized that certain *Smad* genes and/or important transcriptional factors could be involved in the effects of cementogenesis stimulated by MWCNT-COOH. The increased *Runx2* expression after one day of incubation suggested the initiation of cementoblast mineralization. Interestingly, MWCNT-COOH can differently affect the expression of Smads from the same group (*R-Smads*). *Smad2/3* reached the highest expression at Day 3, while Smad1 showed no significant increase. *Smad1* and *Smad2/3* perform essential, but not identical, functions in the process of bone formation. *Smad1*, *2*, *3*, *5* and *8* all belong to the *R-Smads* family, and the structures of *Smad1*, *5* and *8* are very similar [37]. *Smad2/3* serve as substrates for the TGF-β receptors, while *Smad1*, *5* and *8* are substrates for the bone morphogenetic protein (BMP) receptors [38]. Our results demonstrated that MWCNT-COOH can activate the TGF-β signaling pathway and promote differentiation and mineralization in cells. Activations of *Smad2/3* were further confirmed by the increases of the *p-Smad2/3*/*Smad2/3* ratio conducted by western blot. On the other hand, *I-Smads*, namely *Smad6* and *7*, are antagonists of *R-Smad* signaling, which act as competitive inhibitors for *R-Smad* genes. The expression of *Smad7* mRNA was affected by MWCNT-COOH, which did not increase during the first three days of incubation. However, the expression was significantly activated at Day 7. The activated expression of inhibitor TFG-β signaling at the end of cell incubation indicated that MWCNT-COOH may affect the tight regulation of different stages of cementogenesis.

The above analyses confirmed our hypothesis that MWCNTs can enhance gene expression related to differentiation in exposed cementoblasts. The possible mechanism of the enhanced mineralization may be attributed to the up-regulation of key genes at different stages of cementum formation.

## 4. Experimental Section

### 4.1. Preparation of MWCNT-COOH Solution

The MWCNT-COOH used in this work, purchased from Shenzhen Nanotech Port Co., Ltd. in Shenzhen, China, was 20–30 nm in diameter and 1–5 µm in length with a purity of >97%. The MWCNTs were synthesized by the chemical vapor deposition (CVD) method and later functionalized by carboxylation to improve their dispersion in aqueous solutions. The MWCNT-COOH was dispersed in deionized water to reach final working concentrations and sterilized by the UV method for 30 min. All of the MWCNT-COOH solutions were sonicated 20 min for uniform dispersion before they were added to cell culture media [39].

### 4.2. Cell Culture

The OCCM-30, an immortalized murine cementoblast, was provided by Martha J. Somerman (University of Washington, Seattle, WA, USA). These were obtained by isolating root-surface cells from transgenic mouse molars, and all exhibited the same properties of comparable cells found* in vivo* suitable for the present study. The cells were cultured in a high-glucose Dulbecco’s Modified Eagle’s Medium (Hyclone, Logan, UT, USA) supplemented with a 10% FBS and 1% penicillin–streptomycin antibiotic mixture. Cells were seeded at a density of 1 × 10^4^ cells/cm^2^ and incubated at 37 °C with 5% CO_2_. The MWCNT-COOH was added to the media after a 24-h attachment period at concentrations of 5, 15 and 25 µg/mL, and the media were refreshed every 2–3 days during the experiment.

### 4.3. Cell Viability Assay

Using a Cell Counting Kit-8 (CCK-8) assay according to the manufacturer’s procedures (Dojindo, Kumamoto, Japan), the cell growth inhibition rate (IR) of the cultured cells was assessed after they were exposed to MWCNTs for 24, 48 and 72 h. Briefly, the medium was aspirated and replaced by 90 µL fresh serum-free medium with 10 µL water-soluble tetrazolium salts-8 (WST-8) solution per well at the end of each time point and incubated for 2 h at 37 °C. Afterwards, the absorbance of tetrazolium salt (WST-8) was measured at 450 nm using a Micro-plate Reader (VariOskan Flash 3001, Thermo, Waltham, MA, USA). The value of each plate represented the direct correlation with the number of living cells. Values of the measured WST-8 were used to calculate values of IR with Equation (1) [40]:

IR = (1 − (A − B)/(C − B)) × 100
(1)

where A = the value in the treated samples; B = the value in the blank samples; and C = the value in the control samples. This test was done in quadruple and repeated three times.

### 4.4. Assessments of the Material Structure and Cell Growth

Both materials and the materials grown with cells were observed via scanning electron microscopy (SEM). The cells were seeded onto glass slides in a six-well plate and cultured with 5 µg/mL MWCNT-COOH after 24 h of attachment. After 3 days of culture, the samples were rinsed three times with PBS to remove non-adherent cells and then chemically fixed in a 4% paraformaldehyde at room temperature for 20 min. The samples were then dehydrated through an ascending series of ethanol and air. Finally, the samples were coated with Pt-Pd alloys in an ion sputterer, and the morphology of the cells was observed under SEM (FEI Inspect F, Eindhoven, The Netherlands).

### 4.5. Alizarin Red Staining

Alizarin red was used to detect calcium deposits formed by the OCCM-30 cultured with MWCNT-COOH. Briefly, cells were seeded in six-well plates. After 24 h of attachment, the medium was switched to a α-minimal essential medium (α-MEM)-conditioned media with 10% FBS, 50 mg/mL ascorbic acid, 10 mmol/L glycerolphosphate and various concentrations of MWCNTs. After 14 days of culture, the cementoblasts were fixed in 4% paraformaldehyde at room temperature for 20 min and subsequently stained with Alizarin Red S (1% solution).

### 4.6. RNA Isolation and Quantitative Real-Time PCR (qRT-PCR)

After cell incubation for 1, 3 and 7 days, the total RNA was isolated by the TRIzol method, strictly following the manufacturer’s protocol (Invitrogen, Carlsbad, NM, USA). The RNA concentration and purity were assayed at 260 and 280 nm using a spectrophotometer. One microgram of total RNA was reverse transcribed into cDNA using an RNA-PCR kit (Takara, Tokyo, Japan). The resulting cDNA product was used for qRT-PCR. The amplification of alkaline phosphatase (*Alp*), osteocalcin (*Ocn*), bone sialoprotein (*Bsp*), osteopontin (*Opn*), type I collagen (*Col1*), *Runx2*, *Smad1*, *2*, *3*, *6*, *7* and glyceraldehyde 3-phosphate dehydrogenase (*GAPDH*) mRNA was performed with an ABI 7300 real-time PCR system (Applied biosystems 7300, Foster City, CA, USA) using SYBR Green PCR reaction mix (Infinigen, City of Industry, CA, USA). The primer sequences of the tested genes (synthesized by Takara Biological Technology Co., Ltd., Dalian, China) are shown in Table 1. The relative mRNA expression levels were calculated from the threshold cycle (*C*_t_) value and normalized with that of GAPDH using the 2^−∆∆*C*t^ method and presented as fold the change relative to control cells. Each test was done in quadruple and repeated three times.

**Table 1:** Sequences of primers used for real-time PCR analysis.

Genes	Primer Sequence	GenBank Number
*Smad1*	F: 5'-ACCTGCTTACCTGCCTCCTGA-3'	NM_008539.3
	R: 5'-GCAACTGCCTGAACATCTCCTCT-3'	
*Smad2*	F: 5'-TTCACAGACCCATCAAACTCGG-3'	NM_001252481.1
	R: 5'-CTATCACTTAGGCACTCAGCAAACA-3'	
*Smad3*	F: 5'-GACCACCAGATGAACCACAGCA-3'	NM_016769.4
	R: 5'-TAGGAGATGGAGCACCAGAAGG-3'	
*Smad6*	F: 5'-AGGTGTTCGACTTTGAGCGC-3'	NM_008542.3
	R: 5'-CAGGAGGTGATGAACTGTCGC-3'	
*Smad7*	F: 5'-GCTGTCCAGATGCTGTACCTTCC-3'	NM_001042660.1
	R: 5'-GAGTCTTCTCCTCCCAGTATGCC-3'	
*Col1*	F: 5'-GCATAAAGGGTCATCGTGGCT-3'	NM_007742.3
	R: 5'-CCGTTGAGTCCGTCTTTGCC-3'	
*Ocn*	F: 5'-GGACCATCTTTCTGCTCACTCTG-3'	NM_001037939.2
	R: 5'-ACCTTATTGCCCTCCTGCTTG-3'	
*Opn*	F: 5'-TGATGAACAGTATCCTGATGCCA-3'	NM_001204201.1
	R: 5'-CTGCCCTTTCCGTTGTTGTC-3'	
*Bsp*	F: 5'-GAATACGAACAAACAGGCAACG-3'	NM_008318.3
	R: 5'-CATCCTCATAAGCTCGGTAAGTGTC-3'	
*Alp*	F: 5'-ACAACCTGACTGACCCTTCGC-3'	NM_001287172.1
	R: 5'-CAATCCTGCCTCCTTCCACC-3'	
*Gapdh*	F: 5'-ACCACAGTCCATGCCATCAC-3'	NM_008084.2
	R: 5'-TCCACCACCCTGTTGCTGTA-3'	
*Runx2*	F: 5'-GCCACCTCTGACTTCTGCCTCT-3'	NM_001145920.2
	R: 5'-CAGTGAGGGATGAAATGCTTGG-3'	

### 4.7. Western Blot

A western blot was conducted to demonstrate the specific effect of MWCNT-COOH on TGF-β/Smad pathway protein expression. All cells were washed twice and lysed in a lysis buffer solution (0.5% phosphatase inhibitor, 0.1% protease inhibitor and 1 mmol/L phenylmethylsulfonyl fluoride) at 4 °C for 30 min. An equal amount of proteins from each extract was then subjected to Sodium dodecyl-sulfate polyacrylamide gel electrophoresis (SDS-PAGE). After the transfer of SDS-PAGE to nitrocellulose membranes (Invitrogen, Carlsbad, CA, USA), successive incubations with rabbit anti-total Smad2/3, anti-phospho-Smad2/3 (pSmad2/3) and GAPDH primary antibodies (Cell Signaling Technology, Danvers, MA, USA), as well as goat anti-rabbit secondary antibody (Zhongshan Golden Bridge Biotechnology, Beijing, China) were conducted. Immuno-reactive proteins in the samples were then detected using the enhanced chemiluminescence (ECL) system. Membranes were scanned using the ChemiDoc XRS Systems (Bio-Rad, Hercules, CA, USA).

### 4.8. Statistical Analysis

The means ± SDs (standard deviations) were calculated for each array of treatments for preliminary numerical comparisons. Differences between treatment groups were analyzed using one-way ANOVA followed by Newman–Keuls *post hoc* tests. The significance level of the analyses was set at α ≤ 0.05. All data were analyzed with SPSS 19.0 software (SPSS, Chicago, IL, USA).

## 5. Conclusions

MWCNT-COOH enhances cementoblast differentiation and mineralization through the interaction of TGF-β–stimulated Smads. These results suggest the potential for applications of MWCNT-COOH in clinical periodontal regenerative nanomedicine. The present study is preliminary, and further studies on the findings are required.
